# Harnessing nature's biosynthetic capacity to facilitate total synthesis

**DOI:** 10.1093/nsr/nwac178

**Published:** 2022-08-26

**Authors:** Haili Zhang, Ge Liao, Xiaozhou Luo, Xiaoyu Tang

**Affiliations:** Institute of Chemical Biology, Shenzhen Bay Laboratory, China; Institute of Chemical Biology, Shenzhen Bay Laboratory, China; Center for Synthetic Biochemistry, Shenzhen Institute of Synthetic Biology, Shenzhen Institute of Advanced Technology, Chinese Academy of Sciences, China; Institute of Chemical Biology, Shenzhen Bay Laboratory, China

Natural products (NPs) have long piqued the interests of chemists due to their structural complexity. More importantly, evolution has conferred NPs with the ability to interact with biological macromolecules (such as nucleic acids, proteins, carbohydrates and lipids), making them privileged scaffolds for drug discovery. Until now, >50% of clinically approved drugs have been derived directly from NPs or their derivatives [1]. The difficulty in obtaining sufficient materials from their source organisms has been a major challenge in developing novel medicines from NPs. Although synthetic chemistry has arguably advanced to the point at which organic chemists can synthesize practically all NP types, producing most NPs through total synthesis within a reasonable cost and time range remains difficult.

Fundamentally, NPs are genetically encoded and biosynthesized. Over the last few decades, tremendous efforts have been made to elucidate NP biosynthesis, resulting in an expanding enzymatic repertoire available for the synthesis of NPs via synthetic biology. In this perspective, we use three representative examples to illuminate how synthetic biology is emerging in the field of total synthesis and highlight its prospects.

**Figure 1.**
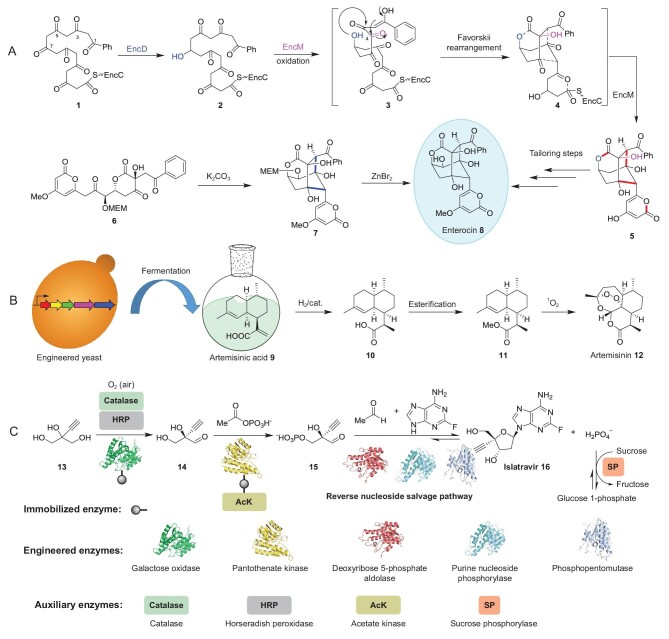
Applications of synthetic biology in total synthesis of complex molecules. (A) Enzymatic and chemical total synthesis of enterocin (**8**). (B) Fermentation and semi-synthesis of artemisinin (**12**). (C) Enzymatic synthesis of the deoxyadenosine analogue islatravir (**16**).

The first example is the bacteriostatic NP enterocin (**8**) from *Streptomyces* (Fig. 1A). The unique tricyclic-caged skeleton of **8** has long fascinated biochemists and chemists, who have worked to solve its biosynthesis and pursue its chemical synthesis, respectively. Moore and colleagues figured out its biosynthetic procedure after over a decade of work [2–4]. Overall, enterocin biosynthesis begins with the assembly of a phenylalanine-derived benzoyl-CoA starter unit and seven malonyl-CoA through a multiprotein enzymatic complex called iterative type II polyketide synthase to produce a highly reactive intermediate **1**. During the chain elongation process, a reductase (EncD) selectively reduced the C7 keto group to yield the dihydrooctaketide (**2**). Oxidation of the C4 methylene group by the flavoprotein EncM triggers a Favorskii-like rearrangement and two intramolecular aldol condensations to form the caged tricyclic core (Fig. 1A). Besides, enterocin was reconstructed *in vitro* by biosynthetic enzymes, indicating the first total enzymatic synthesis of a complex NP [3]. Knowledge of enterocin biosynthesis eventually inspired the development of the first chemical total synthesis of enterocin [5]. The essential step in the synthesis was designed to imitate the two aldol condensations involved in enterocin biosynthesis, resulting in the accomplishment of four of the seven stereogenic centers within the biomimetic reaction cascade (Fig. 1A).

Our second example involves the first-line antimalarial drug, artemisinin (**12**), a sesquiterpene lactone containing an unusual peroxide bridge (Fig. 1B). Since its discovery in the 1970s by Chinese scientists from *Artemisia annua*, artemisinin-based combination therapies have been the central drugs for treating malaria. However, the traditional avenue of obtaining artemisinin (isolated from *A. annua*) depends on the weather and overall harvest, resulting in the shortage of supply and price fluctuations. Aiming to provide a stable source, Keasling and colleagues developed a microbial chemical factory by inserting a few artemisinin biosynthetic genes into *Saccharomyces cerevisiae*, to produce the late-stage artemisinin precursor artemisinic acid (**9**) at 100 mg/L [6]. Then, they employed several synthetic biology strategies, including promoter engineering, codon optimization and functional gene replacement, to enhance the yeast's ability to produce **9**, achieving improved titers of ≤25 g/L [7]. This accomplishment enabled them to establish a scalable synthetic route for easily converting artemisinic acid into artemisinin, with a singlet oxygen source serving as the critical step [8]. This study represents new ground in synergizing the abilities of synthetic biology and chemistry to solve intractable problems in the supply of naturally limited valuable chemicals.

Enzymes are often granted with strict substrate specificity; thus, developing total enzymatic synthesis of unnaturally complex molecules has been a daunting challenge. Ongoing advances in enzyme engineering, along with the ever-increasing enzymology knowledge, have now brought this stretched goal within reach. A terrific example involves a multi-enzymatic cascade for the synthesis of the nucleoside analog islatravir (**16**) [9], an alkyne- and fluorine-containing anti-HIV medication being investigated by Merck. The cascade was designed based on the reversibility of the bacterial nucleoside salvage pathway, which degrades purine 2^′′^-deoxyribonucleosides in nature using a purine nucleoside phosphorylase (PNP), a phosphopentomutase (PPM) and a deoxyribose-5-phosphate aldolase (DERA) (Fig. 1C). To accomplish this incredible task, the authors first engineered these enzymes to bear three non-natural substrates, including a fluorinated base, an alkyne-substituted ribose 5-phosphate and an aldehyde precursor. As the pathway favors digestion, the synthesis struggled significantly with low conversion. This problem was solved by introducing a sucrose phosphorylase to eliminate the inorganic phosphate by-product from the final reaction mixture, thereby promoting the reaction equilibrium towards **16** (Fig. 1C). The authors further established a biosynthetic route for producing 2-ethynylglyceraldehyde-3-phosphate (**15**) for the DERA. Two engineered and immobilized enzymes (a galactose oxidase and pantothenate kinase) and three auxiliary enzymes catalyse the conversion, which starts from simple achiral building block 2-ethynylglycerol (**13**). Together, five engineered enzymes and four auxiliary enzymes were employed to stereoselectively assemble islatravir in three linear steps (51% overall yield). As the process eliminated intermediate purification and protection, the product was produced with fewer steps and a higher yield than the previously reported chemical synthesis (12 steps, 15% overall yield) [10]. This work is a tour de force that indicates a different approach in the synthesis of complex chemicals by harnessing the capacity of biosynthetic enzymes.

As evidenced above, we might be entering a new era in the total synthesis of small molecules with the emergence of synthetic biology. Overall, synthetic biology offers great efficiency, unbeatable precision and indispensable sustainability in chemical production, whereas chemical synthesis provides greater variability and diversity. Therefore, future advances in small molecule synthesis will be driven by a combination of chemistry and biology. Given the complexities of these two fields, it will be difficult for synthetic chemists or biologists to drive the amalgamation alone. We believe that a community effort is urgently needed to transform the field of total synthesis. In return, it will lay the foundation for improving our ability to control the shape and topology of complex small molecules.
